# Follow-up of asymptomatic adult diaphragmatic hernia: should patients with this condition undergo immediate operation? A report of two cases

**DOI:** 10.1186/s40792-016-0220-z

**Published:** 2016-09-09

**Authors:** Ryota Takahashi, Shintaro Akamoto, Mina Nagao, Natsumi Matsuura, Masao Fujiwara, Keiichi Okano, Yasuyuki Suzuki

**Affiliations:** 1 Department of Gastroenterological Surgery, Faculty of Medicine, Kagawa University, Ikenobe, Miki-cho, Kita-gun, Kagawa, 761-0793 Japan; 2 Department of General Thoracic, Breast, and Endocrinological Surgery, Faculty of Medicine, Kagawa University, Miki-cho, Kita, Kagawa, 761-0793 Japan

**Keywords:** Diaphragmatic hernia, Bochdalek hernia, Iatrogenic, Adult, Asymptomatic, Incidentally found, Wait-and-see

## Abstract

**Background:**

Asymptomatic diaphragmatic hernia is generally thought to be rare among adults. We present two different types of asymptomatic diaphragmatic hernia diagnosed with computed tomography (CT) and discuss treatment strategies.

**Case presentation:**

Case 1: A 37-year-old woman was diagnosed with catamenial pneumothorax in the right diaphragm. Partial resection of the diaphragm and lung was performed using a linear stapler. She was asymptomatic after the operation and gave birth 2 years later. After delivery, she experienced recurrent pneumothorax, and CT revealed a right diaphragmatic defect with herniation of a part of the liver into the thorax. An iatrogenic diaphragmatic hernia was diagnosed. There has been no change in the size of the hernia and no symptoms due to the diaphragmatic hernia for more than 3 years after it was diagnosed.

Case 2: A 75-year-old woman was previously diagnosed with rectal cancer and had undergone surgery after chemoradiotherapy. One year after surgery, herniation of a 3 × 1.3-cm section of retroperitoneal fat tissue into the left thoracic cavity was observed incidentally at a follow-up CT and was diagnosed as an adult Bochdalek hernia (BH). We reviewed the patient’s past CT findings and confirmed that the same finding had been present since the first scan. A wait-and-see approach was chosen because there had been no change in the size of hernia, there were no symptoms, the patient was elderly, and there was a high risk of recurrence of the rectal cancer. She has had no symptoms to date, and careful follow-up has been performed.

**Conclusions:**

There are few reports of asymptomatic adult diaphragmatic hernia. Although symptomatic diaphragmatic hernia is generally treated surgically, there are cases in which a wait-and-see approach has been applied, such as our asymptomatic cases.

## Background

There are two main types of diaphragmatic hernia (DH), namely, congenital and acquired [1]. Although neonatal congenital DH is a common, surgically treated disease that is thought of as life-threatening, adult asymptomatic DH is rare. Traumatic DH is sometimes encountered by surgeons in daily practice and usually treated surgically, so most surgeons are interested in whether surgery for adult DH is necessary.

Here, we present two cases of asymptomatic adult DH diagnosed with computed tomography (CT).

## Case presentation

**Fig. 1:**
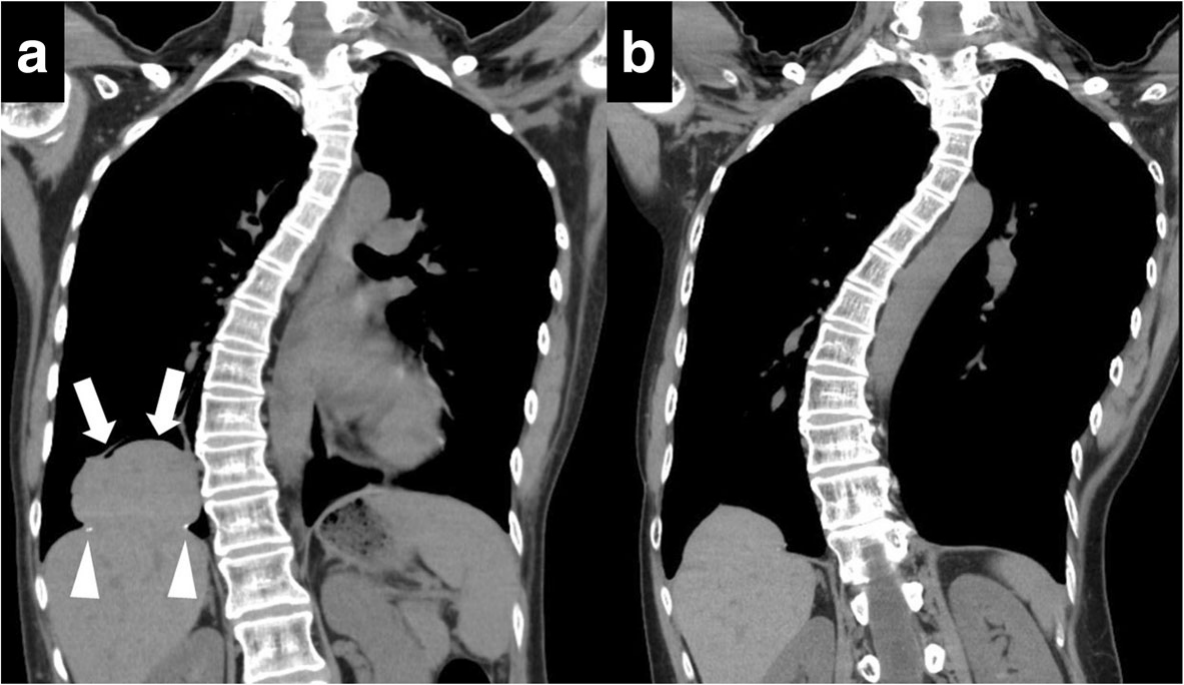
**a** Computed tomography revealed a right diaphragmatic defect with herniation of a part of the liver (6.6 × 4.5 cm) into the thorax (*white arrows*). Surgical staples were observed around the edge of the diaphragmatic defect (*white arrowheads*). **b** Computed tomography finding 3 years after the scan obtained in Fig. 1a. There has been no enlargement of the hernia

Case 1: A 37-year-old woman was diagnosed with catamenial pneumothorax in the right diaphragm. Video-assisted partial resection of the diaphragm and lung was performed using two cartridges of endo staplers (45-3.5). She was asymptomatic after the operation and gave birth 2 years after the operation. After delivery, she complained of chest pain, and CT revealed a recurrent pneumothorax. CT also revealed a right diaphragmatic defect with herniation of a part of the liver (6.6 × 4.5 cm) into the thorax (Fig. 1a). The staples were observed around the edge of the diaphragmatic defect. An iatrogenic diaphragmatic hernia was diagnosed. The pneumothorax was immediately treated with a chest drainage tube. After treatment for pneumothorax, she had no symptoms, and there were no abnormalities based on laboratory examinations. A wait-and-see approach was chosen with the patient’s informed consent. There has been no enlargement of the hernia (Fig. 1b) and no symptoms due to the diaphragmatic hernia for more than 3 years after it was diagnosed.

**Fig. 2:**
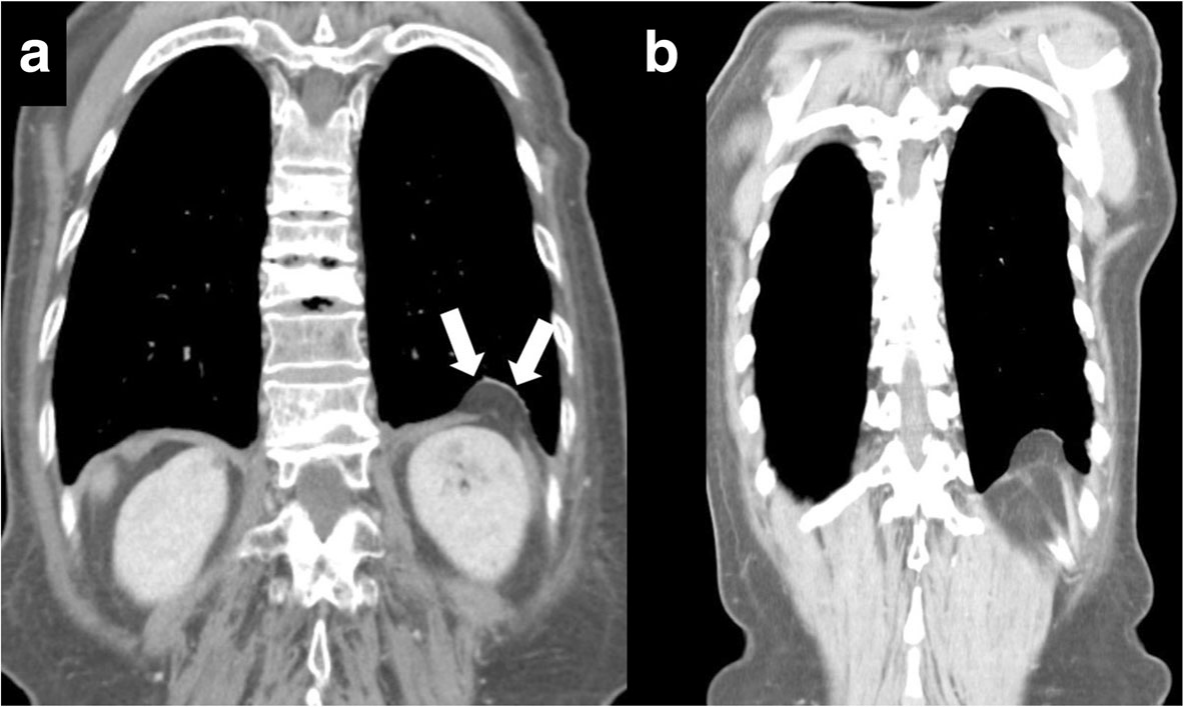
**a** Computed tomography (CT) revealed a defect in the left diaphragm and herniation of retroperitoneal fat tissue (3 × 1.3 cm) into the left thorax (*white arrows*). The size of hernia has not changed compared with the size observed on past CT scans. **b** Past computed tomography finding (1 year and 3 months before the scan obtained in Fig. 2a)

Case 2: A 75-year-old woman with a past medical history of rectal cancer underwent follow-up CT examination, and a left-sided adult Bochdalek hernia (BH) was incidentally observed. She was diagnosed with rectal cancer with invasion into the pelvic cavity at 73 years of age and underwent low anterior resection of the rectum after preoperative chemoradiotherapy. She simultaneously underwent colostomy and resection of the internal iliac vessels, piriformis, coccygeus muscle, and the fifth sacrum. She underwent postoperative adjuvant chemotherapy and had an uneventful course. After 1 year, a follow-up CT scan revealed a defect in the left diaphragm and herniation of the retroperitoneal fat tissue (3 × 1.3 cm) into the left thorax (Fig. 2a). She was diagnosed with a left-sided adult BH. No herniation of any intra-abdominal organs was identified, and laboratory data were unremarkable. We reviewed past CT findings and confirmed the same finding since the first scan; however, the size of hernia has not changed (Fig. 2b). Given that there were no symptoms, that there were no exacerbations on CT, that the patient was an elderly adult, and that there was a high risk of the recurrence of rectal cancer, a wait-and-see approach was chosen. The patient has been symptom-free to date for 1 year and 6 months after diagnosis, and a careful follow-up has been performed.

### Discussion

There are two main types of diaphragmatic hernia (DH), namely, congenital and acquired. Most surgeons associate DH with a disease requiring emergency surgical treatment. Recently, minimally invasive surgery, including thoracoscopic and laparoscopic surgery, has gradually been used to repair adult DH [2, 3]. With respect to the diagnostic modality, multi-slice CT with reformatted coronal and sagittal images is the most effective and useful imaging technique for DH. Because magnetic resonance imaging has a high sensitivity for soft tissue, it may be performed in selected patients, especially for late-presenting DH cases or when the diagnosis is doubtful [4]. We presented two different cases of asymptomatic diaphragmatic hernia diagnosed from CT.

#### Iatrogenic diaphragmatic hernia

Generally, acquired DH is rare, most of the cases are traumatic in origin, and the majority occurs on the left side [5, 6]. Surgical repair is indicated for the treatment of traumatic DH [7]. Iatrogenic DH is a type of acquired DH. Iatrogenic DH is an uncommon postoperative complication and has been reported following transthoracic hiatal hernia repairs, esophagogastrectomies, laparoscopic surgery, hepatectomy, and radiofrequency ablation of liver tumors [1, 8, 9]. The incidence of iatrogenic DH after surgery for the diaphragm is unknown. Tabrizian et al. reported that 5.4 % of 55 patients who underwent hepatic resection with concomitant diaphragmatic resection and repair developed iatrogenic DH [1]. Fragility of the diaphragm due to thermal damage or resection can cause iatrogenic DH [1, 10], and there is no way to prevent such DH from occurring other than to ensure intraoperative diaphragmatic repair. Most of the reported iatrogenic DH cases presented with stomach or intestinal incarceration were symptomatic and required surgical treatment [1, 10]. The anatomic location of the liver minimizes the possibility of a right-sided intestinal or stomach incarceration, and there is no report of the surgical indication for iatrogenic hepatic herniation alone. In our case 1, the viscera involved in the herniation were not part of the digestive tract but a part of the liver, and there were no symptoms or ischemic liver disorder. After delivery of an infant, abdominal pressure would decrease due to the contraction of the uterus, and diaphragmatic muscle relaxation and ligament suppleness would improve [11]. Thus, we thought that this patient could maintain her condition and that the digestive tract would not be incarcerated. There has been no change in size for 3 years. Although most of the reported cases have been treated surgically, a wait-and-see may be preferable in such a case.

#### Bochdalek hernia

The diaphragm develops as part of fetal development, and its development rarely occurs improperly. Some have reported that defects can be caused by certain gene mutations, but the etiology is still unknown [12, 13]. When such a defect exists, the viscera sometimes herniate through it. This anomaly is called congenital DH. The most common type of congenital DH occurs in the posterolateral segment of the diaphragm, which Bochdalek first described and which is called a BH [14, 15]. The prevalence of BH is not clear. This hernia is said to occur in 1 in 2200 to 12,500 live births, and it mostly occurs on the left side of the diaphragm [16–18]. Most patients with symptomatic BH are diagnosed immediately after birth by presenting with severe, life-threatening cardiorespiratory distress. In contrast, symptomatic BH in adults accounts for 10 % of all cases of BH. Various factors are said to be associated with the onset of BH, and they are divided into congenital and acquired cases. The former type occurs when a patient is born with a diaphragm defect but presents no symptoms until he or she is an adult. Patients with no apparent traumas are included in this category. The latter type occurs when factors such as trauma affect a fragile part of the diaphragm. In our case, the patient had no medical history of abdominal surgery or a trauma before she was recognized as having BH, so she was diagnosed with congenital BH.

Mullin et al. previously reviewed 13,138 cases examined with CT, and 22 cases of BH (0.17 %) were found in 1 year; they thus concluded that BH is rare [19]. However, that study was based on a retrospective review of abdominal CT reports using keyword searches. Thus, their review may not represent the true incidence of cases. In fact, there are some recent retrospective reports of multi-detector row CT findings. Kinoshita et al. reported 396 (12.7 %) cases of adult BH out of 3107 patients who underwent CT examination over a period of 7 months, and Osman et al. reported that there were 142 (10.5 %) cases among 1350 patients over a period of 10 months; they concluded that BH in adults is not rare [20, 21]. To detect a small hernia of the thorax, sagittal CT scans are more effective than coronal scans [20]. According to these reports, there may be more cases of adult asymptomatic BH than previously thought. However, it is important to note that most of the reported herniated structures were not digestive tract organs but adipose tissue or omental fat [20, 21]. Although small and asymptomatic BHs do not need to be treated surgically [22], there are no criteria to determine surgical indications for cases of digestive tract hernias. If case 2 had been recognized as BH at the time of the operation for rectal cancer, we do not think that there would have been a need to repair it because there was no herniation of the digestive tract. The wait-and-see approach is more preferable to surgical repair for patients with no symptoms of BH, especially when there is no digestive tract herniation.

## Conclusions

We described two cases of asymptomatic DH that were incidentally found during postoperative CT examination and provided a literature review. Our cases help to illustrate the differences between cases requiring surgery and those for which the wait-and-see approach may be preferable.

## Abbreviations

DH: Diaphragmatic hernia
BH: Bochdalek hernia
CT: Computed tomography
